# The Use of Online Consultation Systems and Patient Experience of Primary Care: Cross-Sectional Analysis Using the General Practice Patient Survey

**DOI:** 10.2196/51272

**Published:** 2024-07-26

**Authors:** Xiaochen Ge, Paul Chappell, Jean Ledger, Minal Bakhai, Geraldine M Clarke

**Affiliations:** 1 Improvement Analytics Unit The Health Foundation London United Kingdom; 2 NHS England London United Kingdom

**Keywords:** general practice, online consultation, patient experience, modern general practice, sociodemographic health inequalities, General Practice Patient Survey, cross-sectional study

## Abstract

**Background:**

NHS England encourages the use of online consultation (OC) systems alongside traditional access methods for patients to contact their general practice online and for practices to manage workflow. Access is a key driver of patients’ primary care experience. The use of online technology and patient experience vary by sociodemographic characteristics.

**Objective:**

This study aims to assess the association between OC system use and patient experience of primary care in English general practice and how that varies by OC system model and practice sociodemographic characteristics (rurality, deprivation, age, and ethnicity).

**Methods:**

We categorized practices as “low-use” or “high-use” according to the volume of patient-initiated contacts made via the OC system. We considered practices using one of 2 OC systems with distinct designs and implementation models—shorter “free text” input with an embedded single workflow OC system (FT practices) and longer “mixed text” input with variation in implemented workflow OC system (MT practices). We used 2022 General Practice Patient Survey data to capture 4 dimensions of patient experience—overall experience, experience of making an appointment, continuity of care, and use of self-care before making an appointment. We used logistic regression at the practice level to explore the association between OC system use and patient experience, including interaction terms to assess sociodemographic variation.

**Results:**

We included 287,194 responses from 2423 MT and 170 FT practices. The proportions of patients reporting positive experiences at MT and FT practices were similar or better than practices nationally, except at high-use MT practices. At high-use MT practices, patients were 19.8% (odds ratio [OR] 0.802, 95% CI 0.782-0.823) less likely to report a good overall experience; 24.5% (OR 0.755, 95% CI 0.738-0.773) less likely to report a good experience of making an appointment; and 18.9% (OR 0.811, 95% CI 0.792-0.83) less likely to see their preferred general practitioner; but 27.8% (OR 1.278, 95% CI 1.249-1.308) more likely to use self-care, compared with low-use MT practices. Opposite trends were seen at FT practices. Sociodemographic inequalities in patient experience were generally lower at high-use than low-use practices; for example, gaps in overall experience between practices with the most and fewest White patients decreased by 2.7 percentage points at MT practices and 6.4 percentage points at FT practices. Trends suggested greater improvements in experience for traditionally underserved groups—patients from urban and deprived areas, younger patients, and non-White patients.

**Conclusions:**

An OC system with shorter free text input and an integrated single workflow can enhance patient experience and reduce sociodemographic inequalities. Variation in patient experience between practices with different sociodemographic characteristics and OC systems underscores the importance of tailored design and implementation. Generalizing results across different OC systems is difficult due to variations in how they are integrated into practice workflows and communicated to patients.

## Introduction

Improving patients’ experience of care has been an important policy focus for the National Health Service (NHS) in England [1]. Patient experience is a key outcome of health care [2,3] and a key component of the quality of care [4,5]. Patient experience is also an important factor in effective treatment outcomes, as satisfied patients are more likely to accept, adhere to, and continue with medication and treatment [6].

Over the past few years, general practices have started to adopt online consultation (OC) systems to support more inclusive and flexible access and delivery of primary care services. OC systems allow patients to use digital channels to contact their general practice, ask health-related questions, report symptoms, submit an administrative query, or receive health advice or help. The system gathers relevant information upfront, either directly from the patient or their caregiver or via care navigators or other practice staff who complete the form on behalf of patients over the phone. Practices can then use this information to understand the patient’s need and respond in the right way by navigating and triaging the requests to the right service or professional [7].

A main driver of change has been the health policy in the English NHS. The 2019 *NHS Long Term Plan* committed to improving patient access to primary care by 2023 or 2024 [8] with a focus on the use of digital routes, particularly through the use of OC systems. The COVID-19 pandemic accelerated the adoption and use of digital access channels and contributed to a rapid growth in the use of OC systems to minimize the risk of exposure to COVID-19 infection for patients and staff [9]. Many practices in England now use OC systems combined with an embedded single workflow where all requests are managed equitably regardless of the route of contact. This model of implementation can support consistent filtering and navigation of requests, enabling fairer and safer allocation of the right type of appointment, correctly matched to the patients’ needs on the first attempt. In 2023, in the context of rising demand and pressures on frontline staff, NHS England set out a commitment to implementing this “modern general practice” approach in the Delivery Plan for Recovering Access to Primary Care [10]. The modern general practice approach is a way of organizing work in general practice to provide more inclusive access and improve the understanding of demand. Using digital tools, practices can better align existing capacity with need through structured information gathering at the point of contact and consistent use of care navigation across all access channels to get patients to the right person or service at the right time. The approach aims to ensure that care is provided safely and equitably (including the continuity of care), optimizing the use of a multiprofessional team and improving the efficiency of practice processes.

There are numerous, constantly evolving OC systems now available for general practices in England with different designs and models of implementation. In particular, differences in request input formats and length, functionality (use of algorithms), workflow, and integration with other software have been found to have different impacts on patient experience [11].

Advancing equality and minimizing health inequalities is a key ambition of the *NHS Long Term Plan*. There is well-documented variation in patient experience of primary care in relation to sociodemographic factors—ethnic minority patients [12,13], younger patients [14], and patients living in the most deprived [15] and urban areas [16] tend to report the least positive experiences. However, there is little evidence on how the shift toward the use of OC systems in general practice will affect sociodemographic inequalities in patients’ experience of primary care.

Given the rapid scale-up and diversity of OC systems, we sought to investigate how patients’ experience of general practice varied between practices with high or low use of an OC system. We examined 2 OC systems with distinct designs and implementation models that are likely to result in different patient experiences. Key differences in the design relate to how information is gathered in a request (input)—either as shorter “free text” structure questions or as a longer “mixed input” of free text and logic-based, multiple-choice questions. Key differences in intended implementation relate to whether the OC system is consistently embedded with a single workflow for managing all patient requests regardless of the access route or not. We also explored changes in gaps in experience between practices with the most and least positive experience in relation to sociodemographic characteristics, including practice location (rural or urban) and deprivation, patient age, and ethnicity profiles. The aim of this research is to inform the design and implementation of OC systems and how they might be used to improve equity in patients’ experience of primary care.

## Methods

### Data and Data Sources

This was a retrospective, cross-sectional study using aggregate practice-level data from the 2022 General Practice Patient Survey (GPPS), a major national survey that captures multiple aspects of patient experiences in local practices in England [17]. Questionnaires are sent to a random sample of patients who are 16 years of age or older and have been continuously registered with their general practice for at least 6 months. Data are weighted to account for some people being more likely to respond than others. This adjusts the data to account for any demographic differences between all eligible patients in the practice and those who respond to the survey. Additional details regarding the sampling, weighting, questionnaire design, and data collection are published elsewhere [18]. Aggregate practice-level data from the 2019 GPPS was used to control for prior patient experience.

We obtained volumes of patient-initiated requests made via an OC system between March 2021 and February 2022 at practices using 1 of the 2 OC systems with key differences in their design and implementation model:

- Free text input with an embedded single workflow OC system (FT OC system): Patients enter their requests using short, structured free text responses [19]. Tick-box questions allow patients to indicate whether they are a patient, parent, or caregiver; their request type as a new medical problem, an existing problem, or any other question; and whether they would like to see a preferred clinician. Practices are encouraged to capture all incoming requests, whether initiated online, in person, or by telephone (and completed on behalf of patients by care navigators or other practice staff), in the OC system to support a model of modern general practice, where there is parity of access and prioritization regardless of the access route [20].
- Mixed text input with variation in implemented workflow OC system (MT OC system): Patients enter their requests using a mix of long free text and logic-based, multiple-choice questions. This questionnaire is longer than the one used in the FT OC system. An algorithm triages and flags response according to the clinical urgency. Patients may then be automatically redirected to urgent or emergency care, for example, to call NHS 111 or go directly to an emergency department. There is variation in implementation models, with some practices managing all requests via a single workflow and others via multiple workflows. For example, requests initiated in person or by telephone may be handled separately from those incoming via the OC system.

Both systems provide patients with clear self-care instructions, the ability to nominate a preferred clinician, and aim to respond to requests within a fixed timeframe. Practices may respond via online or text message, telephone call, or a face-to-face consultation. GPPS and patient-initiated request data were linked at the practice level to publicly available information capturing practice characteristics. These included sociodemographic characteristics of registered patients and practice workforce data from NHS Digital [21], area deprivation from the English Index of Multiple Deprivation [22], rural or urban location from the Office of National Statistics [23], and Care Quality Commission ratings from NHS Digital [24].

### Study Population and Use of the OC System

We included all practices using the MT or FT OC system between March 2021 and February 2022 (hereafter referred to as MT or FT practices, respectively). We determined the number of requests that were initiated online via the OC system per 1000 registered patients per practice per month (usage rate). Practices were categorized as high-use or low-use according to the usage rate over the study period (Figure S1 in Multimedia Appendix 1). This was assumed to be a proxy for whether a practice was fully (high-use) or only partially (low-use) using the OC system for patient-initiated demand. Due to the much larger number of MT practices, we also included a medium-use category for MT practices. Multimedia Appendix 1 presents the practice inclusion criteria and use categorization.

### GPPS Experience Dimensions

We selected 4 dimensions of patient-reported experience of primary care—overall experience, experience of making an appointment, ability to see or speak to a preferred general practitioner (GP; hereafter referred to as the continuity of care), and trying to get information or advice before trying to get an appointment (hereafter referred to as self-care). These dimensions were chosen based on their likelihood of both being impacted by the design of the OC system and where past research has demonstrated their importance in driving overall experience of primary care [25]. Full question wording and details are presented in Table S1 in Multimedia Appendix 1.

### Study Covariates

For each practice, we determined list size; number of full-time equivalent GPs; Care Quality Commission rating; proportion of registered patients by age, gender, ethnicity, and education level; rural or urban practice location; practice deprivation; whether the practice starting using the OC system before or after the COVID-19 pandemic began in March 2020; and prior patient experience scores for each of the patient experience dimensions in 2019. Practice deprivation was categorized according to deprivation score quintile, with quintile 1 representing a practice located in 20% of most deprived areas in England [26]. Practice rural or urban location was determined by Lower Layer Super Output Area Statistics [23].

### Statistical Methods

Multiple-choice responses to the GPPS questions (Table S1 in Multimedia Appendix 1) were summarized as follows. For 3 of the 4 questions with a 5-point ordinal scale response, binomial variables were created by combining the 2 most positive responses together versus the other responses. For example, the overall experience score was measured as the proportion of responders at a given practice reporting either a “very good” or “fairly good” overall experience. For the question relating to the use of self-care before seeking an appointment, binomial variables were created by combining all positive responses indicating that some action had been taken versus the response of “no action taken.”

We used logistic regression [27] to explore the relationship between the use of the OC system and each dimension of patient experience with adjustment for covariates. We standardized continuous variables to ensure they were on the same scale [28]. To explore whether gaps in experience between practices with the most and least positive experience in relation to practice sociodemographic characteristics varied between low-use and high-use MT and FT practices, we tested for an interaction between each sociodemographic characteristic and use level in turn. For interaction analyses, continuous age and ethnicity variables were converted into categorical variables representing different proportions of registered patients older than 65 years of age and of White ethnicity, respectively. Predicted experience scores were calculated by averaging practice characteristics in each interaction group [29] and were used to trace changes in patient experience in low-use and high-use practices by their sociodemographic characteristics.

All analyses were carried out on a secure analysis server at the Health Foundation using R (version 4.0.3; R Core Team) [30]. Data were provided to the research team under a data sharing agreement with NHS England and are not publicly available.

### Sensitivity Analysis

We conducted sensitivity analyses to assess the reliability of our findings. One sensitivity analysis involved weighting negative responses (proportions of patients reporting a “poor” or a “very poor” experience) when rating patient experience. Another sensitivity analysis applied a different set of usage rate thresholds to address the robustness of the current approach used to categorize use.

### Ethical Considerations

The authors declare that this research meets ethical guidelines. The GPPS is designed to give patients the opportunity to give feedback about their experiences of their general practice. The original data collection is approved by the Central Office for Research Ethics Committee (COREC) and carried out by Ipsos MORI for NHS England. Ipsos MORI is a registered and independent survey organization that strictly adheres to the Market Research Society’s ethical code of conduct. The patient-initiated request counts from OC systems were anonymized and not identifiable to the research team carrying out the research. This analysis did not receive an exemption from an institutional review board as its review was not required for this research, which is limited to the secondary use of information previously collected in the course of normal care and where the patients are not identifiable to the research team carrying out the research.

## Results

### Practice Characteristics

A total of 287,164 responses from the 2022 GPPS survey from 2423 MT and 170 FT practices were included in the analysis. The overall response rates at MT and FT practices were 28.3% and 32.1%, respectively. Sociodemographic characteristics of MT and FT practices are broadly comparable to practices in the rest of England (Table 1) with a few notable exceptions—high-use MT practices were less likely to be in the top 20% of most deprived areas or a rural area and more likely to have more registered patients compared with practices nationally; high-use FT practices were also less likely to be in the top 20% of most deprived areas but more likely to be in a rural area and have a greater proportion of patients older than 65 years of age and of White ethnicity compared with practices nationally.

**Table 1:** Patient sociodemographic characteristics of general practices using MT^a^ and FT^b^ OC^c^ systems and English practices nationally (2022).

Characteristic			MT practices						FT practices					National practices (n=6518)
			Low use (n=932)		Medium use (n=825)		High use (n=666)		Low use (n=91)		High use (n=79)			
**Deprivation, n (%)**														
	1 (most deprived)	271 (29.3)		218 (26.8)		146 (22.1)		24 (26.7)		16 (20.5)		1806 (27.78)		
	2	217 (23.4)		170 (20.9)		156 (23.6)		24 (26.7)		23 (29.5)		1501 (23.09)		
	3	174 (18.8)		163 (20.0)		138 (20.9)		14 (15.6)		18 (23.1)		1248 (19.2)		
	4	143 (15.4)		145 (17.8)		109 (16.5)		17 (18.9)		12 (15.4)		1017 (15.64)		
	5 (least deprived)	121 (13.1)		117 (14.4)		112 (16.9)		11 (12.2)		9 (11.5)		929 (14.29)		
**Location, n (%)**														
	Rural	140 (15.2)		92 (11.4)		56 (8.5)		19 (21.1)		20 (25.6)		954 (14.74)		
	Small town	271 (29.4)		325 (40.1)		242 (36.7)		18 (20.0)		22 (28.2)		2516 (38.89)		
	Urban	510 (55.4)		393 (48.5)		361 (54.8)		53 (58.9)		36 (46.2)		3000 (46.37)		
**CQC^d^ rating, n (%)**														
	1	4(0.4)		1(0.1)		3(0.5)		0(0.0)		0(0.0)		29 (0.46)		
	2	48 (5.3)		36 (4.5)		27 (4.1)		3(3.3)		3(4.0)		281 (4.46)		
	3	829 (90.7)		730 (90.9)		603 (92.2)		82 (90.1)		70 (92.1)		5,681 (90.23)		
	4	33 (3.6)		36 (4.5)		21 (3.2)		6 (6.6)		3 (4.0)		305 (4.84)		
Practice list size, median (IQR)			6465 (4294-9174)		8685 (5892-12,126)		9645 (6612-13,666)		8402 (5258-10,906)		8348 (5764-11,899)		8028 (5229-11,710)	
Number of full-time equivalent doctors per 1000 registered patients, median (IQR)			0.49 (0.37-0.66)		0.54 (0.40-0.72)		0.56 (0.42-0.71)		0.54 (0.42-0.71)		0.58 (0.45-0.76)		0.53 (0.40-0.71)	
Registered patients aged >65 years (%), median (IQR)			17.07 (12.61-21.93)		17.98 (13.72-22.17)		17.13 (13.03-21.62)		19.69 (17.44-23.73)		19.89 (17.81-22.78)		18.18 (13.77-22.34)	
**Registered patients by ethnicity group (%), median (IQR)**														
	Asian	4.17 (1.14-13.02)		3.22 (1.24-10.35)		4.33 (1.47-12.86)		1.76 (0.92-5.43)		1.28 (0.87-3.88)		3.52 (1.2-11.21)		
	Black	1.46 (0.29-7.09)		1.11 (0.3-5.39)		1.51 (0.34-7.06)		0.61 (0.31-1.18)		0.53 (0.32-0.93)		1.03 (0.32-4.85)		
	Mixed	1.90 (0.94-3.96)		1.76 (1.00-3.66)		2.02 (1.10-3.93)		1.05 (0.76-1.95)		1.02 (0.77-1.78)		1.72 (0.98-3.47)		
	White	90.85 (64.8-97.27)		93.22 (74.55-97.16)		90.56 (68.42-96.74)		96.31 (90.68-97.86)		96.89 (92.77-97.86)		92.66 (75.14-97.25)		
	Other	0.55 (0.18-2.43)		0.50 (0.18-1.91)		0.58 (0.21-2.31)		0.27 (0.14-0.72)		0.23 (0.15-0.39)		0.42 (0.17-1.49)		
Registered male patients (%), median (IQR)			49.36 (48.66-50.30)		49.15 (48.50-50.14)		49.21 (48.61-50.13)		49.31 (48.75-49.71)		49.09 (48.64-50.01)		49.24 (48.65-50.02)	
Registered patients with higher education (%), median (IQR)			11.33 (10.03-12.24)		11.78 (10.67-12.61)		11.81 (10.62-12.67)		12.11 (11.68-12.67)		12.03 (11.35-12.90)		11.70 (10.67-12.52)	

^a^MT: mixed text input with variation in implemented workflow online consultation system.

^b^FT: free text input with an embedded single workflow online consultation system.

^c^OC: online consultation.

^d^CQC: Care Quality Commission.

### Association Between Use of the OC System and Patient Experience

The proportions of patients reporting positive experiences at MT and FT practices were similar or better than practices nationally, except at high-use MT practices (Table 2). High-use MT practices had lower proportions of patients reporting a positive experience than both low-use MT practices and practices nationally across all experience dimensions except self-care. Conversely, high-use FT practices had higher proportions of patients reporting a positive experience than both low-use FT practices and practices nationally across all experience dimensions except self-care. For example, 68.8% and 78.2% of respondents at high-use MT and FT practices, respectively, reported a good overall experience, compared with 72.4% of practices nationally. For self-care, 66% and 61.6% of respondents at high-use MT and FT practices, respectively, reported use of self-care before making an appointment, compared with 61.5% of practices nationally.

**Figure 1:**
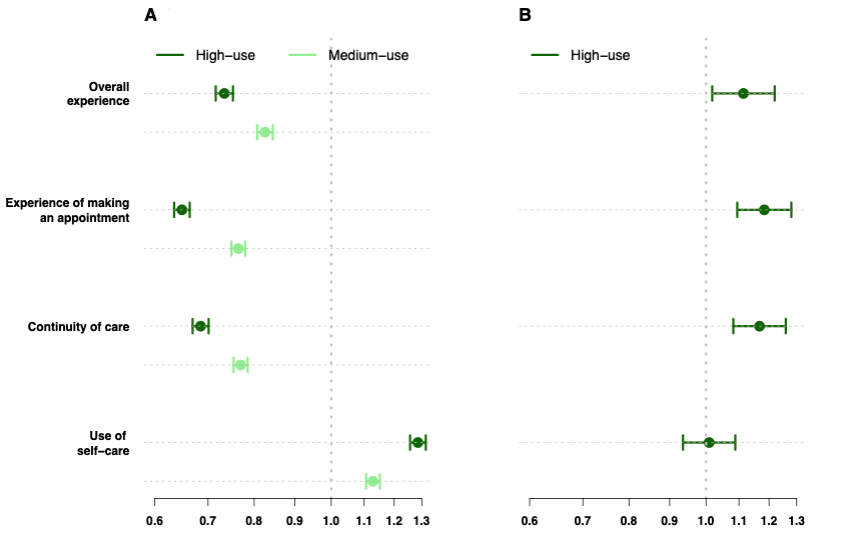
Association between patient experience and use of an OC system at (A) MT practices and (B) FT practices. FT: free text input with an embedded single workflow online consultation system; MT: mixed text input with variation in implemented workflow online consultation system; OC: online consultation.

These trends are confirmed after adjusting for underlying differences between the practices (Figure 1 and Table S2 in Multimedia Appendix 1) in regression modeling. At MT practices, high use of the OC system is associated with poorer overall experience (odds ratio [OR] 0.8, 95% CI 0.78-0.82; *P*<.001), experience of making an appointment (OR 0.75, 95% CI 0.74-0.77; *P*<.001), and continuity of care (OR 0.81, 95% CI 0.79-0.83; *P*<.001) but better use of self-care (OR 1.28, 95% CI 1.25-1.31; *P*<.001), compared with low use. At FT practices, high use of the OC system is associated with better overall experience (OR 1.23, 95% CI 1.13-1.32; *P*<.001), experience of making an appointment (OR 1.31, 95% CI 1.23-1.4; *P*<.001), and continuity of care (OR 1.28, 95% CI 1.20-1.37; *P*<.001) but not with use of self-care (OR 0.99, 95% CI 0.93-1.06; *P*=.87), compared with low use.

**Table 2:** Numbers of included responses and weighted national patient experience in England at general practices using MT^a^ and FT^b^ OC^c^ systems and English practices nationally (2022).

Dimension	MT practices, n (%)				FT practices, n (%)		National practices, n (%)
	Low use	Medium use	High use	Low use		High use	
Good overall experience	81,184 (75.5)	96,289 (72.8)	86,837 (68.8)	9406 (72.7)		9410 (78.2)	710,421 (72.4)
Good overall experience of making an appointment	76,210 (61.9)	90,215 (56.1)	81,315 (49.9)	8788 (57.1)		8807 (66.2)	666,553 (56.2)
Continuity of care	34,225 (44.4)	37,433 (37.6)	31,125 (33.8)	3567 (39.6)		3422 (44.7)	274,407 (38.2)
Self-care	75,787 (58.9)	89,645 (62.2)	80,964 (66)	8756 (62.3)		8732 (61.6)	662,857 (61.5)

^a^MT: mixed text input with variation in implemented workflow online consultation system.

^b^FT: free text input with an embedded single workflow online consultation system.

^c^OC: online consultation.

### Sociodemographic Inequalities

**Figure 2:**
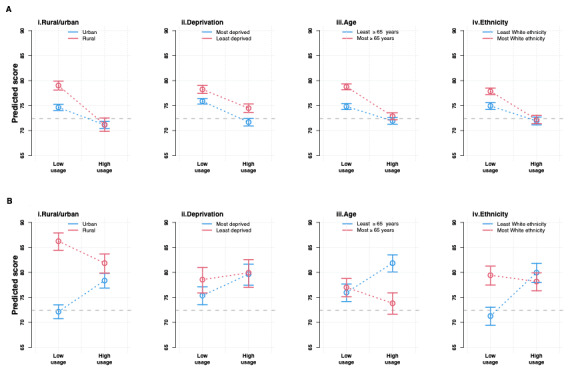
Trends in inequalities in overall patient experience in (A) MT practices and (B) FT practices. The y-axis presents adjusted predictions of the proportion of patients reporting a good response for the overall experience in primary care. Average estimates with 95% CIs are presented for each practice sociodemographic group. The absolute change between low-use and high-use practices can be seen visually in the figure and corresponds to the values presented in Table 3 column 1. Groups represented in red or blue are those that traditionally report the least or most challenges in their experience of primary care. FT: free text input with an embedded single workflow online consultation system; MT: mixed text input with variation in implemented workflow online consultation system.

Including an interaction in our multivariable model allowed us to predict average reported experience scores at low-use and high-use MT and FT practices in different sociodemographic groups (Figure 2, Table 3, and Figures S2-S4 in Multimedia Appendix 1). We have denoted in blue and red the sociodemographic groups that traditionally experience the worst and best experience of primary care, respectively.

For all patient experience dimensions other than self-care, (1) predicted patient experience at low-use MT and FT practices was poorest for the practices in the traditionally most challenged sociodemographic groups—namely, those in urban and most deprived areas and with the greatest proportion of patients aged younger than 65 years and of non-White ethnicity; (2) declines in predicted patient experience between low-use and high-use MT practices were more pronounced for practices in the least challenged sociodemographic groups—namely, those in rural and least deprived areas and with the greatest proportion of patients aged older than 65 years and of White ethnicity; and (3) improvements in average patient experience between low-use and high-use FT practices were chiefly driven by practices in the most challenged sociodemographic groups.

Improvements in the use of self-care between low-use and high-use MT practices tended to be most pronounced at practices in the least challenged sociodemographic groups. There was no significant sociodemographic variation in the use of self-care between low-use and high-use FT practices.

Table 3 summarizes how the difference in average experience between the most and least positive practice sociodemographic groups changed between low-use and high-use practices. A surfeit of significant negative estimates overwhelmingly suggests that sociodemographic inequalities are narrower at high-use than at low-use practices. For example, the difference in overall experience between rural and urban practices is 4.3 (*P*<.001) and 10.6 (*P*<.001) percentage points smaller at high-use compared with low-use MT and FT practices, respectively. There were some notable nonnegligible exceptions to this trend in relation to age—differences in experience between practices with the greatest and least proportions of patients older than 65 years of age were wider at high-use compared with low-use practices for continuity of care (3.1 percentage points, *P*<.001) at MT practices and for overall experience (7.0 percentage points, *P*<.001) and experience of making an appointment (5.0 percentage points, *P*<.001) at FT practices. Results were robust to sensitivity analyses.

**Table 3:** Changes in inequalities in patient experience between high-use and low-use practices using MT^a^ and FT^b^ OC^c^ systems^d^.

Practices and inequalities				Overall experience		Experience of making an appointment		Continuity of care		Use of self-care
**MT practices**										
	**Location (rural or urban)**									
		Change, n	–4.31		–3.01^e^		–2.54^e^		–1.26^e^	
		Interaction *P* value	<.001^f^		<.001^f^		<.001^f^		<.001^f^	
	**Deprivation**									
		Change, n	0.43		–0.65		–0.06		0.02	
		Interaction *P* value	<.001^f^		<.001^f^		<.001^f^		<.001^f^	
	**Age**									
		Change, n	–3.03		–2.55		3.12		–2.21	
		Interaction *P* value	<.001^f^		<.001^f^		<.001^f^		<.001^f^	
	**Ethnicity**									
		Change, n	–2.64		–2.01^e^		–4.57		–2.2	
		Interaction *P* value	.003^f^		<.001^f^		<.001^f^		.28	
**FT practices**										
	**Location (rural or urban)**									
		Change, n	–10.63		–13.03		3.93^e^		–2.44	
		Interaction *P* value	<.001^f^		<.001^f^		<.001^f^		.06	
	**Deprivation**									
		Change, n	–2.89		–1.74^e^		0.04^e^		2.23^e^	
		Interaction *P* value	.03^f^		.05		.01^f^		.1	
	**Age**									
		Change, n	6.99^1^		5.04^e^		–2.34		0.03^e^	
		Interaction *P* value	<.001^f^		<.001^f^		<.001^f^		.3	
	**Ethnicity**									
		Change, n	–6.39^e^		–5.51^e^		5.76^e^		2.59	
		Interaction *P* value	<.001^f^		<.001^f^		<.001^f^		.42	

^a^MT: mixed text input with variation in implemented workflow online consultation system.

^b^FT: free text input with an embedded single workflow online consultation system.

^c^OC: online consultation.

^d^The interaction *P* values presented in this table can be interpreted as a measure of the strength of evidence that inequalities in patient experience changed between low-use and high-use practices for each sociodemographic characteristic and for each experience outcome. The summary measure (change) is the difference in predicted experience between the absolute difference in predicted experience between the most and least positive practice sociodemographic groups at low-use practices and the equivalent measure at high-use practices. Negative values indicate that inequalities between the most and least positive practice groups have narrowed or improved; the converse is applicable for positive values. For all dimensions other than self-care, low-use practices experiencing the most challenge are generally those in urban and most deprived areas, with the greatest proportion of patients aged younger than 65 years and from ethnic minority groups. For self-care, the opposite is true, with low-use practices in rural and least deprived areas, and with the greatest proportion of patients aged 65 years or older and of White ethnicity experiencing the most challenge at low-use practices.

^e^The inequality has flipped with the most challenged sociodemographic group at low-use practices now the least challenged sociodemographic group at high-use practices.

^f^Significant results (*P*<.05).

## Discussion

### Patient Experience and Use of an OC system

Practice use of an OC system was associated with substantial differences in the patient-reported experience of primary care across dimensions of the overall experience, the experience of making an appointment, continuity of care, and self-care at the practice level. These results varied by the type of OC system and by practice sociodemographic factors providing important new insights into optimal OC system design and implementation.

Opposing effects on patient experience between low-use and high-use MT practices compared with FT practices suggests that differences in the design and implementation model of each OC system are critical.

### Design

The MT OC system uses multiple-choice questions for inputting requests, which has been shown to increase patient workload and decrease patient satisfaction. Problems include the length of time taken to complete [31-33], confusing navigation [34], or being redirected to services that do not address the patient’s needs [35]. In the latter case, patients may amend their responses to achieve a different outcome [31]. Conversely, the FT OC system uses shorter free text for inputting requests, enabling patients to express their requests in their own words and potentially reduce the time for completion. These differences in how requests are input might help explain the contrasting effects on patient overall experience and experience of making an appointment between low-use and high-use MT and FT practices. Use of self-care is better at high-use than at low-use MT practices but is not associated with use at FT practices. Self-care options may be more apparent or readily accessible on MT OC systems, or alternatively, algorithms used by MT OC systems may be more likely to guide patients toward self-care alternatives. Although careful consideration needs to be given to the design of self-care options in an OC system to assess their appropriateness for each patient, these findings suggest that OC systems could be effectively used to encourage self-care.

### Implementation

Both MT and FT OC systems allow patients to nominate a preferred clinician, which is expected to improve continuity of care [36]. However, only patients at FT practices reported enhanced continuity at high-use compared with low-use practices. FT OC systems offers a model of digitally supported information gathering and care navigation, where all incoming requests, whether by telephone or online, are processed through a single workflow allowing for consistent understanding of needs, navigation, and triage. Greater variability in workflow models at MT practices may offer some insights into why reported continuity is worse at high-use than at low-use MT practices, but more research would be needed to confirm this theory [36].

### Inequalities in Patient Experience

The association between patient use of an OC system and patient experience of primary care varied by practice-level sociodemographic factors. Practices in urban and most deprived areas, as well as those with the greatest proportion of patients aged younger than 65 years and from ethnic minority groups, tended to benefit the most from high use of the OC system. Notably, these are the groups that typically report the worst experience of primary care [12,14,16]. Here, we found that these groups reported greater improvements (FT practices) or less pronounced declines (MT practices) in experience in all dimensions except self-care at high-use compared with low-use practices than their counterparts—namely, those in rural and least deprived areas and with the greatest proportion of patients aged older than 65 years and of White ethnicity.

These findings suggest that the FT OC system design and indicated implementation model is more likely that the MT OC system design and indicated implementation model to address the challenges faced by those patients who traditionally report the worst experience of primary care. Factors that may explain differences in experience across different sociodemographic groups are explained in the following sections.

### Work or Caring Commitments

OC systems provide patients with flexible ways to contact their local practices, allowing them to avoid the inconvenience and cost of traveling to their practice or waiting on the telephone [37]. The improved access may be of greater benefit to people from the most deprived communities who are more likely to find it difficult to take time away from work or caring commitments [38].

### Language Barriers

Research shows that shorter question sets and free text entry can be easier and faster for patients to answer and align best with their speaking and communication styles [11]. This may be particularly important for patients for whom English is not their first language and who may feel more confident or at ease sending an online request, where they can take more time to express themselves or can receive help via online translation systems or from relatives or friends [39].

### Population Health and the Demand-Capacity Gap Within a Practice

People from ethnic minority backgrounds or those residing in disadvantaged areas shoulder a higher burden of health problems compared with people of White ethnicity or those living in wealthier areas [40]. Practices in deprived areas tend to be overrepresented by patients from ethnic minority groups [40] and are underfunded and underresourced relative to need [41,42]. Hence, a greater demand-capacity gap might make getting an appointment or having sufficient time during the appointment more difficult at practices in deprived areas or with a high proportion of patients from ethnic minority backgrounds. If an OC system helps practices to enhance their care navigation and better align capacity with needs more efficiently and effectively, it has the potential to have a greater impact on these challenged practices than on well-funded and well-resourced practices.

### Digital Inclusion and Adoption

People in rural areas often face greater challenges accessing fast and reliable digital technologies [43] and have a greater proportion of older patients compared with people living in urban areas. Older patients also have greater health care needs [44] and may be slower to adopt new technologies [45] or find it more difficult or frustrating to use digital systems compared with younger patients [46]. The greater need for care combined with less familiar access routes to care may lead to a poorer experience at high than at low-use practices for older patients and those living in rural areas. Research also shows that both nondigital and digital users can benefit from modern general practice, with older patients and those with greater clinical needs being prioritized as well as faster access being provided for older patients, young children, those with new presentations, and those with more complex care needs [47]. Hence, improving support for the implementation and use of digital tools as part of modern general practice has the potential to improve patient experience for older patients over time [48].

### Patient Expectations

Patient expectations and perceptions prior to experiencing care are theorized to be a mediator of patient satisfaction [49]. Individuals from ethnic minority backgrounds often have lower expectations of general practice compared with those of White ethnicity [50]. High-use practices with large proportions of patients from ethnic minority backgrounds generally report better experiences (except for self-care) compared to high-use practices with mostly White patients. This might suggest that the expectations of patients from ethnic minority backgrounds are easier to meet, rather than indicating a truly better experience.

The finding that the continuity of care was better at high-use FT practices compared with both low-use FT practices and practices nationally may offer further evidence that the approach to navigation and triage indicated by the FT OC system can enable greater continuity of care. Similarly, self-care was better at high-use MT practices compared with both low-use MT practices and practices nationally for all sociodemographic groups. This may suggest that the ability to incorporate options for self-care in an OC system can be useful in addressing some of the barriers or challenges to self-management known to be greater in some populations than others, for example, people experiencing socioeconomic deprivation [51].

### Narrowing of Inequalities

Our findings show that differences in reported patient experience between the most and least challenged practice groups tend to be smaller at high-use than at low-use practices. This may support leveling up of access across sociodemographic groups and so contribute to more inclusive access. It may also suggest that practices that have fully implemented an OC system may have invested more time and resources in raising awareness and supporting its use. More work is required to focus on design and implementation across the 2 systems and to understand in greater depth the variation between different patient cohorts and why benefits are not realized for practices using MT OC systems.

### Limitations

There were 4 main limitations of our study. First, we only captured total counts of patients using an OC system at practice, not who or how patients were using it, nor whether all patients responding to the survey had actually used them. We also could not account for variations in how they are integrated into practice workflows, how they are communicated to patients, and how their data are collected. These factors may have reduced the power to detect effects and to disentangle which features of design and implementation are responsible for the effects we see. Second, the analysis was conducted at the practice rather than the patient level, so it was not possible to make conclusions about the impact of OC systems on individual patients or to adjust at the patient level for patient comorbidities, which may be correlated with patient experience and the sociodemographic characteristics considered.

Third, as with most patient experience surveys [52], some practices included in this analysis have a relatively low survey response rate, which may introduce systematic bias into the data. However, the GPPS data are weighted to account for differences between all patients at a practice and the subset that takes part in the survey. This mitigates the issue of a low response rate and other studies have reported little evidence to support concerns related to nonresponse bias in analyses using the GPPS survey [53,54]. Finally, responses could not restrict all patients’ interaction with practices within a given timeframe. Hence, recall bias because of systematic differences between the ability of respondents to accurately recall details of their most recent GP experience may be introduced.

### Conclusions

The OC systems have the potential to increase overall experience of general practice, experience of making an appointment, continuity of care, and use of self-care and to reduce sociodemographic disparities in access to care. However, stark differences in reported patient experience at practices using different types of OC systems suggest that careful consideration as to how the systems are designed and implemented, particularly in relation to local population needs and demographics, is required to achieve this. Generalizing and evaluating impacts across different OC systems is difficult due to variations in how they are integrated into practice workflows, how they are communicated to patients, and their constant evolution

## Appendix

Multimedia Appendix 1 Online consultation system use, General Practice Patient Survey 2022 questions and available answers for selected domains, trends in inequalities in overall experience of making an appointment, trends in inequalities in the ability to see or speak to a preferred general practitioner (continuity of care), and trends in inequalities in seeking any kind of information or advice before trying to make an appointment (self-care utilization).

## Abbreviations

COREC: Central Office for Research Ethics Committee
FP practices: practices using the free text input with an embedded single workflow online consultation system
FT OC system: free text input with an embedded single workflow online consultation system
GP: general practitioner
GPPS: General Practice Patient Survey
MT practices: practices using the mixed text input with variation in implemented workflow online consultation system
MT OC system: mixed text input with variation in implemented workflow online consultation system
NHS: National Health Service
OC: online consultation
OR: odds ratio

## Data Availability

Data on patient use of online consultation systems were provided to the research team under a data sharing agreement with NHS England and are not publicly available due to commercial sensitivities. The General Practice Patient Survey (GPPS) data sets analyzed during this study are freely available [17].
