# Supplementary material for: The Use of Online Consultation Systems and Patient Experience of Primary Care: Cross-Sectional Analysis Using the General Practice Patient Survey
Source: J Med Internet Res. 2024 Jul 26;26:e51272. doi: 10.2196/51272 (PMC11316161; doi:10.2196/51272)
Supplement: Multimedia Appendix 1 [file jmir_v26i1e51272_app1.docx]

**Multimedia Appendix 1**

# Practice inclusion criteria and categorization by online consultation system usage rate

For each practice, we calculated the online consultation (OC) system usage rate (hereafter referred to as the usage rate) as the number of patient-initiated contacts made via the OC system per 1,000 registered patients per practice per month. In order to reduce bias, we removed practices that had no recorded contacts in any single month of the study period. These practices may be turning the OC system on and off which may result in an underestimate of their average usage rage over the study period. Practices with fewer than 6 months of data were also removed. We also excluded atypical practices with fewer than 750 registered patients in total, or with fewer than 500 registered patients per full-time GP.

W used a data driven approach to categorize practices that accounted for the different usage rate profiles of the two OC systems (Supplementary Figure 1). Due to the smaller sample size of practices using the FT OC system (FT practices), FT practices were categorized in only 2 groups as either “low-use” or “high-use” according to the median usage rate threshold of 220 patient-initiated requests per 1,000 registered patients per month; practices using the MT OC system (MT practices) were categorised into 3 groups as either “low-use”, “medium-use” or “high-use” according to usage rate thresholds of 20 and 45 contacts per 1,000 registered patients per month.

**Figure S1**. Distribution of monthly online consultation system usage rate at practices using MT^a^ and FT^b^ OC^c^ systems. FT practices are classed as “low-use” or “high-use” practices according to whether their usage rate is to the left or right of the green line, respectively. MT practices are categorised as “low-use”, “medium-use” or “high-use” according to whether their usage rate falls to the left of the red line, between the red and green line or to the right of the green line, respectively.


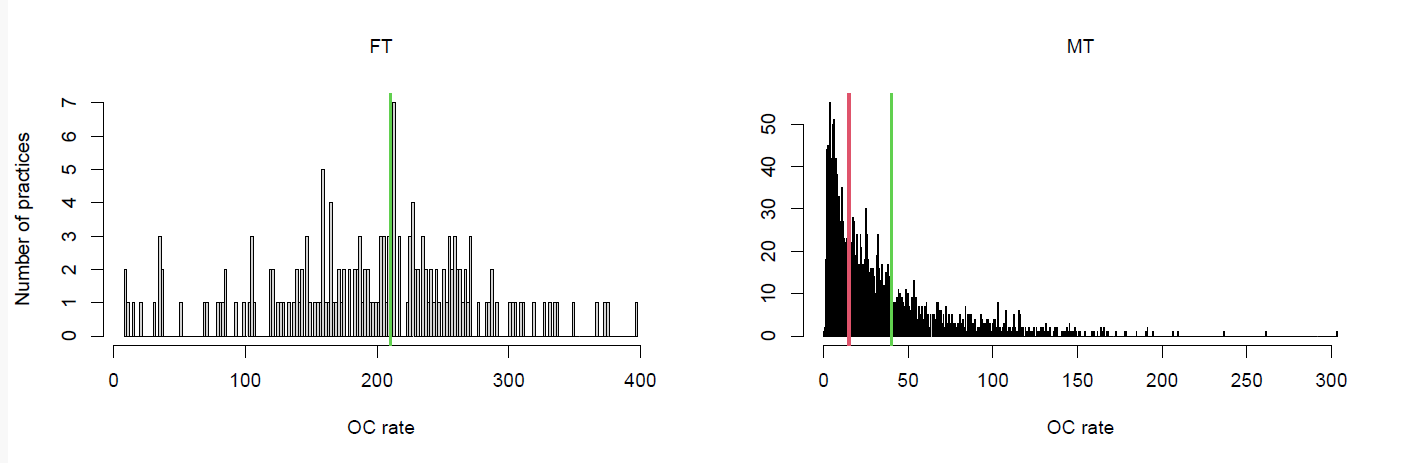


^a^MT: mixed-text input with variation in implemented workflow OC system.

^b^FT: free-text input with an embedded single workflow OC system.

^c^OC: online consultation.

# Supplementary Tables

**Table S1.** GPPS 2022 Survey questions and available answers for selected domains

| Domain | Question | Answer options | Metric |
| --- | --- | --- | --- |
| Overall experience | Q32. Overall, how would you describe your experience of your GP practice? | - Very good - Fairly good - Neither good nor poor - Fairly poor - Very poor | Proportion of responses reporting ‘very good’ or ‘fairly good’ |
| Experience of making an appointment | Q21. Overall, how would you describe your experience of making an appointment? | - Very good - Fairly good - Neither good nor poor - Fairly poor - Very poor | Proportion of responses reporting ‘very good’ or ‘fairly good’ |
| Continuity of care | Q8. How often do you see or speak to your preferred GP when you would like to? | - Always or almost always - A lot of the time - Some of the time - Never or almost never - I have not tried | Proportion of responses reporting ‘Always or almost always’ or ‘A lot of the time’ |
| Use of self-care | Q10. Before you tried to get this appointment, did you do any of the following? | - Used an online NHS service (including NHS 111 online) - Used a non-NHS online service, or looked online for information - Spoke to a pharmacist - Tried to treat myself / the person I was making this appointment for (for example with medication) - Called an NHS helpline, such as NHS 111 - Contacted or used another NHS service - Asked for advice from a friend or family member - Tried to get information or advice elsewhere (from a non-NHS service) - I did not try to get information or advice | Proportion of responses reporting any answer apart from ‘I did not try to get information or advice’ |

**Figure S2.** Trends in inequalities in overall experience of making an appointment at practices using MT^a^ and FT^b^ OC^c^ systems.


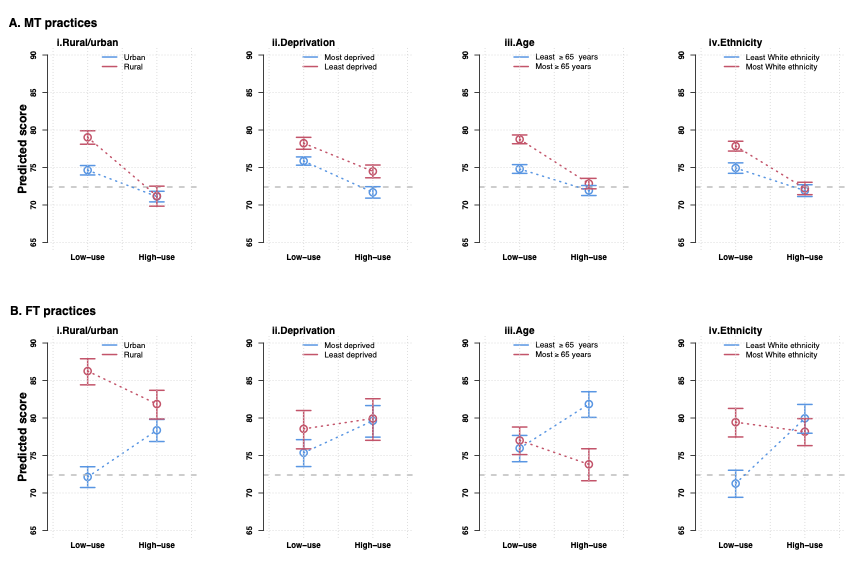


The y-axis presents adjusted predictions of the proportion of patients reporting a good response for the overall experience of making an appointment in primary care. Average estimates with 95% confidence intervals are presented for each practice socio-demographic group. The absolute change in the predicted response between low-use and high-use practices can be seen visually on each plot and corresponds to the values presented in Table 3 column 1. Groups represented in red (blue) are those that traditionally report the least (most) challenges in their experience of primary care.

^a^MT: mixed-text input with variation in implemented workflow OC system.

^b^FT: free-text input with an embedded single workflow OC system.

^c^OC: online consultation.

**Figure S3.** Trends in inequalities in the ability to see or speak to a preferred GP when you want to (continuity of care) at practices using MT^a^ and FT^b^ OC^c^ systems.


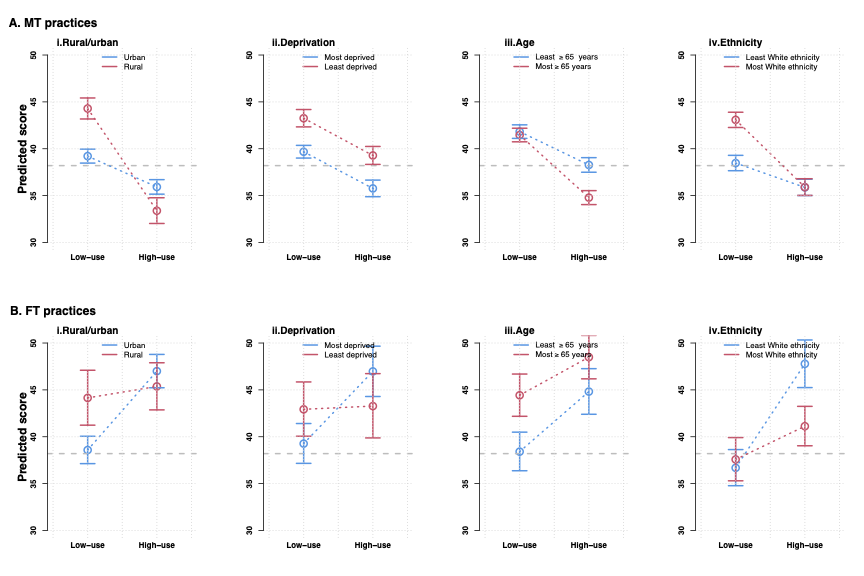


The y-axis presents adjusted predictions of the proportion of patients reporting a good continuity of care. Average estimates with 95% confidence intervals are presented for each practice socio-demographic group. The absolute change in the predicted response between low-use and high-use practices can be seen visually on each figure and corresponds to the values presented in Table 3 column 1. Groups represented in red (blue) are those that traditionally report the least (most) challenges in their experience of primary care.

^a^MT: mixed-text input with variation in implemented workflow OC system.

^b^FT: free-text input with an embedded single workflow OC system.

^c^OC: online consultation.

**Figure S4.** Trends in inequalities in seeking any kind of information or advice before trying to make an appointment (use of self-care) at practices using MT^a^ and FT^b^ OC^c^ systems.


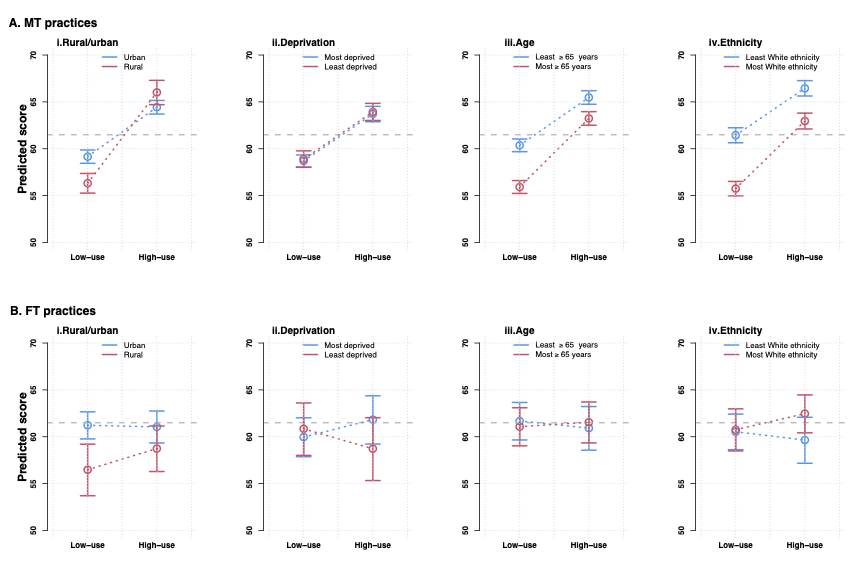


The y-axis presents adjusted predictions of the proportion of patients reporting use of self-care. Average estimates with 95% confidence intervals are presented for each practice socio-demographic group. The absolute change in the predicted response between low-use and high-use practices can be seen visually on each figure and corresponds to the values presented in Table 3 column 1. Groups represented in red (blue) are those that traditionally report the least (most) challenges in their experience of primary care.

^a^MT: mixed-text input with variation in implemented workflow OC system.

^b^FT: free-text input with an embedded single workflow OC system.

^c^OC: online consultation.

**Table S2.** Regression results for association between dimensions of patient experience and practice use of an online consultation system.

1. **Practices using the MT^a^ online consultation system**

|  | **Overall experience** | | **Experience of making an appointment** | | **See or speak to preferred GP (continuity of care_** | | **Use of self-care before making an appointment** | |
| --- | --- | --- | --- | --- | --- | --- | --- | --- |
| **Medium-use** | 0.866[0.846-0.886] | <.001 *** | 0.829[0.812-0.846] | <.001 *** | 0.851[0.833-0.869] | <.001 *** | 1.125[1.103-1.148] | <.001 *** |
| **High-use** | 0.802[0.782-0.823] | <.001 *** | 0.755[0.738-0.773] | <.001 *** | 0.811[0.792-0.83] | <.001 *** | 1.278[1.249-1.308] | <.001 *** |
| **CQC^b^ rating 2** | 1.104[0.955-1.276] | 0.18 | 1.071[0.93-1.233] | 0.34 | 1.079[0.914-1.273] | 0.37 | 1.123[0.976-1.293] | 0.11 |
| **CQC^b^ rating 3** | 1.289[1.121-1.482] | <.001 *** | 1.194[1.042-1.367] | 0.01 * | 1.171[0.997-1.375] | 0.05 | 1.122[0.98-1.285] | 0.1 |
| **CQC^b^ rating 4** | 1.525[1.313-1.771] | <.001 *** | 1.34[1.162-1.546] | <.001 *** | 1.194[1.011-1.41] | 0.04 * | 1.09[0.946-1.256] | 0.23 |
| **Late adopter of online consultation system** | 0.967[0.944-0.991] | 0.01 * | 0.950[0.929-0.971] | <.001 *** | 0.946[0.925-0.968] | <.001 *** | 0.99[0.969-1.012] | 0.37 |
| **% registered patients with higher education** | 1.016[1.004-1.029] | 0.01 * | 1.040[1.028-1.051] | <.001 *** | 1.004[0.993-1.015] | 0.49 | 1.016[1.005-1.027] | 0.01 * |
| **% registered male patients** | 1.043[1.028-1.058] | <.001 *** | 1.043[1.03-1.056] | <.001 *** | 1.037[1.023-1.051] | <.001 *** | 1.008[0.995-1.02] | 0.24 |
| **Registered patients** | 0.928[0.919-0.937] | <.001 *** | 0.914[0.906-0.923] | <.001 *** | 0.931[0.921-0.941] | <.001 *** | 1.055[1.045-1.065] | <.001 *** |
| **No. of full-time equivalent GPs/1000 registered patients** | 1.075[1.064-1.086] | <.001 *** | 1.049[1.04-1.058] | <.001 *** | 1.013[1.003-1.022] | 0.01 * | 0.977[0.969-0.986] | <.001 *** |
| **Small town** | 1.012[0.976-1.049] | 0.52 | 1.009[0.978-1.041] | 0.56 | 0.993[0.963-1.025] | 0.68 | 1.042[1.011-1.073] | 0.01 * |
| **Urban** | 0.884[0.831-0.941] | <.001 *** | 0.944[0.893-0.998] | 0.04 * | 0.919[0.867-0.974] | <.001 *** | 1.028[0.974-1.086] | 0.31 |
| **% registered patients aged older than 65 years** | 1.035[1.015-1.054] | <.001 *** | 1.034[1.017-1.051] | <.001 *** | 0.999[0.982-1.017] | 0.91 | 0.943[0.928-0.959] | <.001 *** |
| **% registered patients with White ethnicity** | 1.064[1.044-1.084] | <.001 *** | 1.024[1.006-1.042] | 0.01 * | 1.065[1.046-1.084] | <.001 *** | 0.929[0.913-0.946] | <.001 *** |
| **Deprivation quintile 2** | 1.01[0.982-1.039] | 0.47 | 0.994[0.969-1.02] | 0.67 | 1.022[0.995-1.05] | 0.11 | 1.021[0.995-1.047] | 0.12 |
| **Deprivation quintile 3** | 0.987[0.957-1.018] | 0.41 | 0.997[0.97-1.026] | 0.85 | 1.036[1.006-1.067] | 0.02 * | 1.015[0.987-1.043] | 0.3 |
| **Deprivation quintile 4** | 0.995[0.961-1.03] | 0.76 | 0.992[0.962-1.024] | 0.64 | 0.999[0.967-1.032] | 0.96 | 1.043[1.012-1.076] | 0.01 * |
| **Deprivation quintile 5** | 1.066[1.026-1.106] | <.001 *** | 1.021[0.988-1.056] | 0.21 | 1.089[1.053-1.127] | <.001 *** | 1.025[0.992-1.06] | 0.14 |
| **Experience in 2019** | 1.375[1.36-1.39] | <.001 *** | 1.441[1.427-1.456] | <.001 *** | 1.602[1.586-1.618] | <.001 *** | 1.052[1.043-1.062] | <.001 *** |

1. **Practices using the FT^c^ online consultation system**

|  | **Overall experience** | | | **Experience of making an appointment** | | **See or speak to preferred GP (continuity of care_** | | **Use of self-care before making an appointment** | |
| --- | --- | --- | --- | --- | --- | --- | --- | --- | --- |
| **High-use** | | 1.225[1.135-1.324] | <.001 *** | 1.313[1.227-1.404] | <.001 *** | 1.284[1.202-1.371] | <.001 *** | 0.995[0.933-1.061] | 0.87 |
| **CQC^b^ rating 3** | | 2.002[1.672-2.397] | <.001 *** | 2.005[1.673-2.402] | <.001 *** | 1.171[0.942-1.457] | 0.16 | 0.785[0.656-0.94] | 0.01 * |
| **CQC^b^ rating 4** | | 3.044[2.32-3.995] | <.001 *** | 3.2[2.509-4.08] | <.001 *** | 1.331[1.027-1.725] | 0.03 * | 0.731[0.584-0.916] | 0.01 * |
| **Late adopter of online consultation system** | | 0.946[0.86-1.04] | 0.25 | 0.948[0.873-1.029] | 0.2 | 0.995[0.919-1.077] | 0.9 | 0.969[0.897-1.046] | 0.42 |
| **% registered patients with higher education** | | 0.933[0.896-0.971] | <.001 *** | 0.993[0.956-1.031] | 0.71 | 0.991[0.956-1.027] | 0.62 | 1.038[0.999-1.078] | 0.05 |
| **% registered male patients** | | 1.006[0.958-1.057] | 0.81 | 0.973[0.931-1.017] | 0.22 | 1.026[0.982-1.071] | 0.25 | 1.006[0.964-1.049] | 0.79 |
| **Registered patients** | | 0.996[0.958-1.036] | 0.85 | 0.993[0.96-1.028] | 0.7 | 1.005[0.97-1.042] | 0.77 | 1.034[0.997-1.072] | 0.07 |
| **No. of full-time equivalent GPs/1000 registered patients** | | 1.161[1.116-1.209] | <.001 *** | 1.051[1.015-1.088] | <.001 *** | 0.953[0.922-0.985] | <.001 *** | 0.955[0.925-0.986] | <.001 *** |
| **Small town** | | 0.616[0.535-0.709] | <.001 *** | 0.619[0.547-0.701] | <.001 *** | 0.826[0.732-0.931] | <.001 *** | 1.252[1.114-1.407] | <.001 *** |
| **Urban** | | 0.581[0.504-0.668] | <.001 *** | 0.577[0.511-0.652] | <.001 *** | 0.937[0.834-1.052] | 0.27 | 1.147[1.024-1.285] | 0.02 * |
| **% registered patients aged older than 65 years** | | 0.902[0.831-0.979] | 0.01 * | 0.918[0.854-0.987] | 0.02 * | 1.022[0.951-1.097] | 0.56 | 0.987[0.922-1.057] | 0.71 |
| **% registered patients with White ethnicity** | | 1.098[1.04-1.158] | <.001 *** | 1.007[0.959-1.057] | 0.79 | 0.984[0.937-1.033] | 0.52 | 0.947[0.903-0.994] | 0.03 * |
| **Deprivation quintile 2** | | 1.022[0.926-1.128] | 0.67 | 1.008[0.923-1.102] | 0.86 | 0.971[0.887-1.063] | 0.53 | 0.979[0.897-1.07] | 0.64 |
| **Deprivation quintile 3** | | 0.948[0.841-1.069] | 0.38 | 0.973[0.875-1.082] | 0.61 | 0.945[0.851-1.049] | 0.29 | 0.998[0.901-1.105] | 0.97 |
| **Deprivation quintile 4** | | 1.005[0.889-1.136] | 0.94 | 0.952[0.854-1.062] | 0.38 | 0.952[0.856-1.058] | 0.36 | 1.098[0.988-1.221] | 0.08 |
| **Deprivation quintile 5** | | 1.119[0.966-1.295] | 0.13 | 1.044[0.92-1.184] | 0.51 | 1.035[0.916-1.169] | 0.58 | 0.971[0.861-1.095] | 0.63 |
| **Experience in 2019** | | 1.345[1.289-1.403] | <.001 *** | 1.423[1.369-1.48] | <.001 *** | 1.478[1.424-1.535] | <.001 *** | 1.032[0.997-1.068] | 0.07 |

^a^MT: mixed-text input with variation in implemented workflow OC system.

^b^CQC: Care Quality Commission

^c^FT: free-text input with an embedded single workflow OC system.
